# Novel neural pathways targeted by GLP-1R agonists and bariatric surgery

**DOI:** 10.1007/s00424-024-03047-3

**Published:** 2024-12-07

**Authors:** Mohammed K. Hankir, Thomas A. Lutz

**Affiliations:** 1 Institute of Veterinary Physiology, University of Zurich, Zurich, Switzerland; 2 School of Biochemistry and Immunology, Trinity College Dublin, Dublin, Ireland

**Keywords:** Obesity, GLP-1, Physiology, Pharmacology, Bariatric surgery, RYGB, VSG

## Abstract

The glucagon-like peptide 1 receptor (GLP-1R) agonist semaglutide has revolutionized the treatment of obesity, with other gut hormone-based drugs lined up that show even greater weight-lowering ability in obese patients. Nevertheless, bariatric surgery remains the mainstay treatment for severe obesity and achieves unparalleled weight loss that generally stands the test of time. While their underlying mechanisms of action remain incompletely understood, it is clear that the common denominator between GLP-1R agonists and bariatric surgery is that they suppress food intake by targeting the brain. In this Review, we highlight recent preclinical studies using contemporary neuroscientific techniques that provide novel concepts in the neural control of food intake and body weight with reference to endogenous GLP-1, GLP-1R agonists, and bariatric surgery. We start in the periphery with vagal, intestinofugal, and spinal sensory nerves and then progress through the brainstem up to the hypothalamus and finish at non-canonical brain feeding centers such as the zona incerta and lateral septum. Further defining the commonalities and differences between GLP-1R agonists and bariatric surgery in terms of how they target the brain may not only help bridge the gap between pharmacological and surgical interventions for weight loss but also provide a neural basis for their combined use when each individually fails.

## Introduction

Deciding on when, where, and what to eat can preoccupy us a great deal. The modern-day environment is laden with sensory cues on our screens, radios, and billboards that frequently encourage us to consume energy-dense, highly processed foods which, over time, can lead to overeating and weight gain [1]. For decades, this trend seemed to be insurmountable contributing to an obesity pandemic that places an enormous burden on our collective health due to obesity’s association with various conditions including type 2 diabetes, cardiovascular disease, metabolic dysfunction-associated steatotic liver disease (MASLD), and certain types of cancer to name just a few [2]. Fortunately, the tide seems to have finally turned, and we are now witnessing an obesity drug revolution with the glucagon-like peptide 1 receptor (GLP-1R) agonist semaglutide spearheading the way [3]. Other gut hormone-based drugs have recently been introduced to the clinic or are still under clinical development and promise to be even more effective than semaglutide, such as the dual GLP-1R and glucose-dependent insulinotropic peptide receptor (GIPR) agonist tirzepatide [4] and the triple GLP-1R, GIPR, and glucagon receptor (GR) agonist retatrutide [5]. While the underlying mechanisms for how these gut hormone-based drugs cause such pronounced weight loss remains an area of intensive investigation, it is clear that they all ultimately target the brain to suppress food intake [6]. This was first suggested for the GLP-1R agonists exendin-4 and liraglutide when it was shown that the suppression of food intake that they cause upon systemic administration is prevented or attenuated when the GLP-1R antagonist exendin-9 is centrally administered in rats [7] and when central GLP-1Rs are deleted in mice [8]. In this Review, we discuss similar preclinical studies that present novel concepts in the neural control of food intake and body weight, with reference to endogenous GLP-1, GLP-1R agonists, and bariatric surgeries like Roux-en-Y gastric bypass (RYGB) and vertical sleeve gastrectomy (VSG), which remain the most effective treatments for severe obesity [9]. Notably, because RYGB and VSG enhance the release of endogenous GLP-1 from enteroendocrine cells due to accelerated nutrient passage through the gut along with enhanced luminal bile acid signaling [10–13], this gut hormone was originally widely thought to contribute to their effects on food intake and body weight [14]. Studies using rodent models cast doubt on the single role of GLP-1 [15–19], although this does not preclude the possibility of common neural mechanisms of action of bariatric surgery with GLP-1R agonists, which we will show target distinct neural pathways to endogenous GLP-1. Indeed, endogenous GLP-1 and GLP-1R agonists appear to have differential access to the brain due to factors such as stability, transportability, and diffusivity [20]. Accordingly, unlike exendin-4 and liraglutide, the suppression of food intake caused by peripheral GLP-1 is attenuated by peripheral but not central administration of exendin-9 [21]. We will take a bottom-up approach starting with the peripheral nervous system and then progress up the brainstem through to the hypothalamus and finish at non-canonical brain feeding centers such as the zona incerta (ZI) and lateral septum.

## The vagus nerve

The vagus nerve of the parasympathetic nervous system richly innervates the gastrointestinal tract where it controls various digestive processes ranging from gastric acid secretion to peristalsis [22]. Based mainly on receptor transcript expression in the nodose ganglia, where the cell bodies of vagal afferents reside, and nerve transection studies, endogenous gut hormones including ghrelin [23], cholecystokinin (CCK) [24, 25], GLP-1 [26], and peptide YY 3–36 (PYY_3–36_) [27] are thought to control food intake via their interaction with vagal afferents in a paracrine manner. This has received support in more recent studies with GLP-1R agonists [7, 28] and PYY_3–36_ [29], provided that they were administered at lower (more physiological) doses. However, with the development of sophisticated techniques that track and manipulate the activity as well as characterize the connectivity of genetically defined vagal afferents, we now have a more nuanced view of how this sensory cell type detects and controls the intake of food [30]. For example, GLP-1R-expressing vagal afferents have been shown to form intraganglionic laminar endings (IGLEs) mainly in the wall of the stomach/upper duodenum in mice [31, 32], such that they are selectively activated by gastric/duodenal stretch and CCK, but unexpectedly, not by exendin-4 [32]. Accordingly, the acute appetite-suppressing effects of liraglutide and the latest GLP-1R agonist iteration semaglutide (but not CCK) are preserved when GLP-1R-expressing vagal afferents are inhibited or selectively ablated [33, 34]. In line with these findings, deletion of GLP-1Rs in paired-like homeobox 2b (PHOX2B)-expressing neurons, which mark all vagal sensory afferents, minimally affects the acute appetite suppressing effects of exendin-4 and only partially prevents the weight loss caused by chronic treatment with the GLP-1R agonist dulaglutide [35]. On the other hand, GPR65-expressing vagal afferents form mucosal endings in duodenal villi, such that they are selectively activated by duodenal infusion of nutrients [32]. Notably, activating GLP-1R-expressing vagal afferents robustly suppresses food intake, while activating GPR65-expressing vagal afferents has a modest effect [31]. Taken together, these findings suggest that gastrointestinal distension as opposed to intestinal nutrient sensing also plays an important role in promoting satiation [31]. In line with this idea, activating oxytocin receptor (OXR)-expressing vagal afferents, which also form IGLEs in the muscular wall of the small intestine in mice, robustly suppresses food intake [31]. Thus, ways of artificially causing or mimicking gastrointestinal distension could potentially promote a negative energy balance in the long term. Indeed, RYGB, which causes marked gastrointestinal distension due to how it forces food passage through the reconfigured gastrointestinal tract, leads to the same central pattern of neuronal activation as activating GLP-1R-expressing and OXR-expressing vagal afferents in mice [31, 36, 37], while vagotomy partially prevents the appetite suppression and weight loss caused by RYGB in rats [38].

## Spinal afferents

Sensory information emanating from the gastrointestinal tract also reaches the central nervous system by way of the dorsal horn of the spinal cord where primary sensory neurons of the splanchnic nerve terminate [33]. While vagal afferents have received the bulk of the attention in the peripheral neural control of food intake and body weight, an increasing number of studies suggest that spinal afferents play a similar role [33]. For example, akin to GLP-1R-expressing vagal afferents, wingless-related integration site 1 (WNT1)-expressing spinal afferents densely innervate the muscular wall of the ileum and large intestine [37]. Interestingly, inhibiting WNT1-expressing spinal afferents increases food intake [37], suggesting a physiological role for this sensory cell type in suppressing food intake. Notably, while deletion of GLP-1Rs in WNT1-expressing neurons does not affect basal food intake and body weight, the acute appetite–suppressing effects of exendin-4 and chronic weight–lowering effects of dulaglutide are diminished [35]. These findings suggest that spinal afferents also seem to play a major role in mediating the effects of GLP-1R agonists on energy balance.

**Fig. 1:**
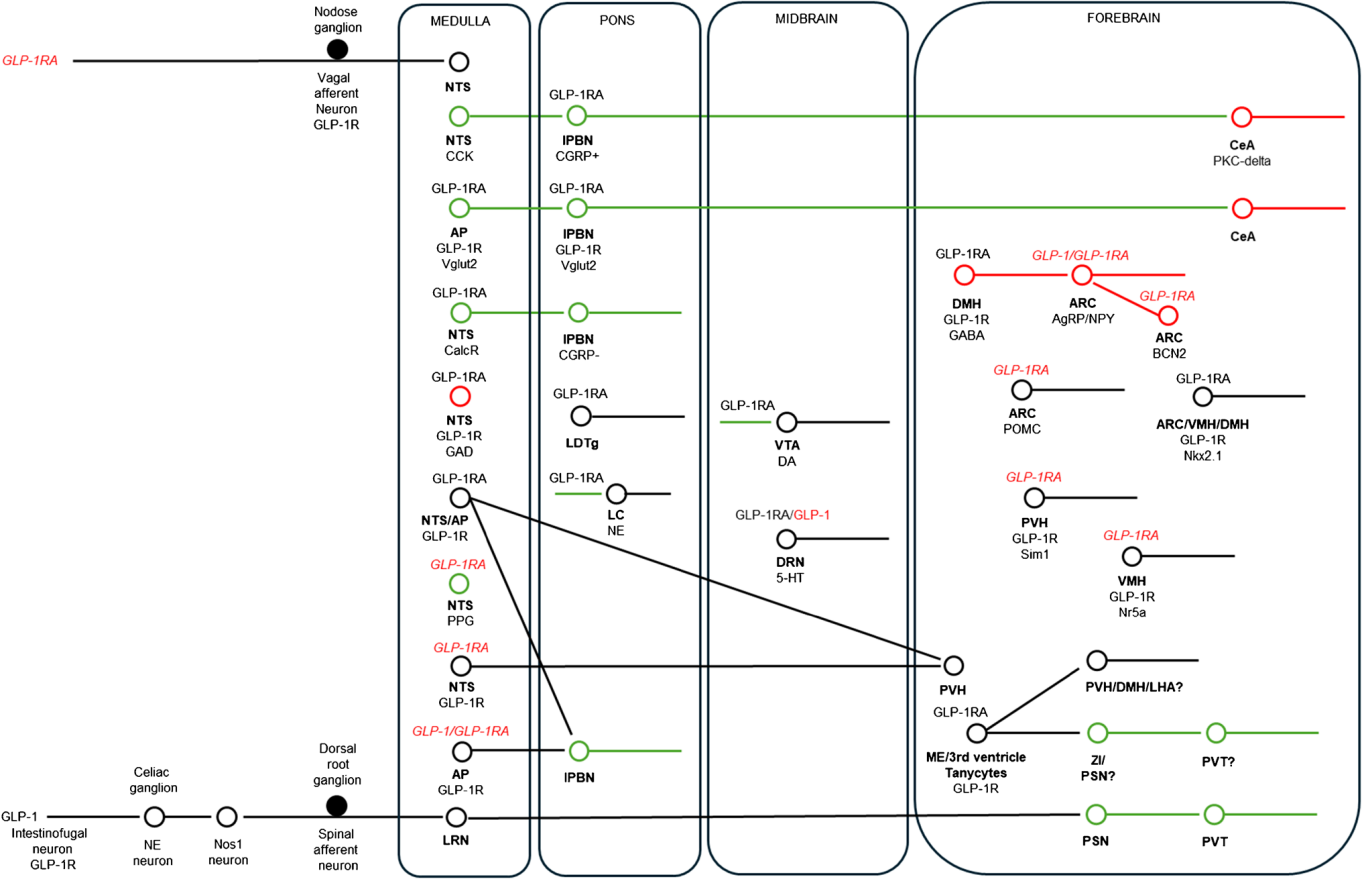
Wiring diagram for GLP-1R agonist-mediated appetite suppression and weight loss. This schematic summarizes peripheral and central neural pathways directly or indirectly targeted by endogenous GLP-1 hormone and systemically administered GLP-1R agonists (GLP-1RA) exendin-4, liraglutide, and/or semaglutide. Text in red italics highlights regions where GLP-1 and GLP-1RAs do not exert their effects based on recording, deletion, inhibition, and/or ablation approaches. Neurons in red are GABAergic, and neurons in green are glutamatergic. In the LC and VTA, GLP-1RA agonists are thought to increase excitatory input onto postsynaptic neurons via presynaptic GLP-1Rs on glutamatergic neuronal terminals

Another example of the control of food intake by spinal afferents comes from a comprehensive study on endogenously released GLP-1 [39]. Because enteroendocrine cells are electrically excitable and fire action potentials [40, 41], they can be artificially activated by chemogenetics or optogenetics like neurons [42, 43]. Activating GLP-1-expressing enteroendocrine cells in the ileum in this manner as well as ileal infusion of picomolar concentrations of GLP-1 (thereby mimicking endogenous release) causes marked gastric dilation and suppression of food intake in mice, which is prevented by ileal infusion of exendin-9 [39]. Interestingly, GLP-1Rs are expressed in intestinofugal neurons that innervate the stomach via the celiac ganglion [39]. Accordingly, ablating pre- and post-ganglionic sympathetic intestinofugal neurons prevents the effects of ileal GLP-1 on stomach dilation and food intake [39]. Conversely, activating pre- and post-ganglionic sympathetic intestinofugal neurons recapitulates the effects of ileal GLP-1 on stomach dilation and food intake [39]. Further, ablating spinal afferents, but not vagal afferents, prevents the inhibitory effects of ileal GLP-1 and artificial stomach dilation on food intake [39]. These findings suggest that rather than only acting in a simple paracrine manner to suppress food intake, endogenously released GLP-1 causes stomach dilation via intestinofugal neurons, which in turn is sensed by spinal afferents innervating the stomach and then transmitted to higher centers via the spinal cord as part of a complex neural arc (Fig. 1). They also provide a salient example of how contemporary neuroscientific techniques have allowed for the mechanistic study of gut-brain communication at an unprecedented level of detail.

## The medulla of the caudal hindbrain

The nucleus tractus solitarius (NTS) of the medulla in the caudal hindbrain is the site where vagal afferents terminate. In rodents, the NTS lies either side of and is heavily interconnected with the area postrema (AP) [44, 45]. Because AP neurons are not fully shielded by a blood–brain barrier [46], the AP/NTS is considered to be the first site of integration of hormonal and neural signals pertaining to acute energy status. Notably, while AP/NTS neurons express GLP-1Rs [3, 47], inhibiting GLP-1R-expressing AP neurons does not prevent the appetite-suppressing effects of endogenously released GLP-1 [39], likely because of low amounts of GLP-1 reaching these neurons due to rapid GLP-1 degradation by the enzyme dipeptidylpeptidase IV (DPPIV) [48]. Nevertheless, AP/NTS neurons seem to be a major target of GLP-1R agonists which are more stable in the bloodstream [49, 50]. Indeed, systemically administered, fluorescently labeled liraglutide and semaglutide gain access to the AP/NTS [51, 52], and administration of exendin-4 into the NTS reduces food intake in rats [53]. Moreover, combined (but not individual) ablation of GLP-1R-expressing neurons in the AP and NTS prevents the appetite-suppressing effects of chronic systemic exendin-4 and semaglutide as well as the weight-lowering effects of chronic systemic semaglutide in mice [34]. On the other hand, inhibiting GLP-1R-expressing GABAergic neurons or deleting GLP-1Rs in the NTS alone is sufficient to partially prevent the appetite-suppressing and/or weight-lowering effects of chronic systemic liraglutide in rats [54]. While the latter findings are also at odds with work showing that GLP-1Rs in glutamatergic rather than GABAergic neurons mediate the effects of chronic systemic liraglutide on energy balance in mice [55], they provide important insight into the neurochemical and neuroanatomical identity of a neuronal network targeted by GLP-1R agonists (Fig. 1). A contribution of NTS astrocytes to the appetite suppression caused by acute GLP-1R agonists also seems possible from evidence showing (1) expression of GLP-1Rs in this cell type, (2) functional responses of astrocytes to exendin-4 and liraglutide, and (3) negation of appetite suppression from administration of exendin-4 into the NTS when astrocyte metabolism is selectively inhibited in this region [56].

A major issue with clinically approved GLP-1R agonists like liraglutide and semaglutide is that they cause nausea and vomiting, which hampers patient compliance [3, 57]. Thus, a burgeoning question in the obesity drug field is whether weight-lowering drugs target separate aversive or non-aversive pathways in the brain to suppress food intake. In this context, activating GLP-1R-expressing neurons in the AP induces aversion [34, 58], while in the NTS, this does not seem to be the case [34]. Moreover, GLP-1Rs in the AP are both necessary and sufficient for the aversion caused by systemic exendin-4 [58] and necessary for the aversion caused by systemic semaglutide [34]. Remarkably, while inhibiting GLP-1R-expressing neurons in the AP prevents the aversion caused by exendin-4 and semaglutide, the chronic inhibitory effects of the two GLP-1R agonists on food intake and body weight are preserved [34, 58]. Similarly, ablating the AP does not prevent the acute appetite suppression caused by systemic exendin-4 [59] and liraglutide [54] in rats. These results suggest that GLP-1R agonists can theoretically be designed to achieve appetite suppression without causing nausea by bypassing GLP-1R-expressing neurons in the AP. Theoretically, one way this can be achieved is by conjugating GLP-1R agonists to a ligand that binds to a receptor exclusively expressed in the NTS. A similar approach has successfully been applied with the selective NMDA receptor antagonist MK-801, with striking effects on food intake and body weight in mice [60].

Within the caudal NTS are two important, non-overlapping subpopulations of neurons that form an integral part of a well-characterized hindbrain feeding circuit. These neurons either express the neurotransmitter norepinephrine (NE) or the neuropeptide CCK and are monosynaptically connected to calcitonin gene–regulated peptide (CGRP)-expressing neurons in the lateral parabrachial nucleus (lPBN) of the pons [61], which are in turn monosynaptically connected to protein kinase C (PKC)-delta-expressing neurons in the central nucleus of the amygdala (CeA) in the forebrain [62, 63], thus forming a tripartite functional unit. Accordingly, refeeding after a fast activates NE-expressing/CCK-expressing NTS neurons [61], CGRP-expressing lPBN neurons [63], and PKC-delta-expressing CeA neurons [62]. Similarly, activating NE-expressing/CCK-expressing NTS neurons [61], CGRP-expressing lPBN neurons [63] or PKC-delta-expressing CeA neurons reduces meal size [62]. This hindbrain feeding circuit is often referred to as an aversive pathway since activating CCK-expressing NTS neurons [64] and CGRP-expressing lPBN neurons [63] causes aversion, with the latter being required for the aversive effects of systemic lithium chloride (LiCl) in mice [65]. In the context of GLP-1R agonists, systemic exendin-4 robustly activates CGRP-expressing lPBN neurons, and its chronic weight-lowering effects are attenuated by their silencing [63]. While not shown, this is most likely mediated by activation of GLP-1Rs in upstream CCK-expressing NTS neurons by exendin-4 since NE-expressing NTS neurons do not appreciably express GLP-1Rs [66]. Notably, both systemic liraglutide and semaglutide treatments robustly activate AP/NTS, lPBN, and CeA neurons [51, 55] through GLP-1Rs expressed in glutamatergic, but not in GABAergic, neurons [55], possibly contributing to their aversive effects. Indeed, GLP-1R-expressing AP neurons, but not GLP-1R-expressing NTS neurons, project to the CGRP-expressing lPBN neurons to cause aversion [34].

As for CCK, GLP-1 is also expressed as a neuropeptide in glutamatergic NTS neurons [67] that inhibit food intake when activated [68]. These neurons send widespread projections to brain regions that highly express GLP-1Rs [66, 69], where administration of GLP-1R agonists reduces food intake and GLP-1R antagonists increase food intake such as the bed nucleus of the stria terminalis (BNST) as a notable example [70] (Fig. 1). The latter suggests a physiological role for GLP-1-expressing NTS neurons in promoting a negative energy balance; however, both their inhibition and ablation fail to have a major effect on baseline food intake and body weight in mice suggesting otherwise [71, 72]. Indeed, GLP-1R knockout mice do not develop obesity [73] although postembryonic deletion of GLP-1Rs in the paraventricular nucleus of the hypothalamus (PVH) leads to weight gain due to increased food intake [72]. Despite not being absolutely necessary for the control of energy balance, GLP-1-expresssing NTS neurons do seem to mediate stress-induced hypophagia and satiation associated with inordinately large meals [71]. Interestingly, ablating GLP-1-expressing NTS neurons fails to prevent the appetite-suppressing effects of systemic liraglutide and semaglutide in mice [74], which is consistent with the lack of GLP-1R expression in these cells [75, 76]. Considering that systemic GLP-1R agonists and central GLP-1 appear to target different central nervous system pathways, these treatments would be expected to additively reduce food intake, which proved to be the case [74].

Based on single-cell RNA sequencing data, a separate subset of NTS neurons, beyond those expressing NE, CCK, and GLP-1, has consistently been identified as glutamatergic calcitonin receptor (CalcR)-expressing neurons in the AP/NTS [67, 77]. These neurons overlap with NE-expressing, but not CCK-expressing, NTS neurons [78], and their activation suppresses food intake without causing aversion [78]. Consistently, CalcR-expressing NTS neurons project to non-CGRP-expressing lPBN neurons [78]. Moreover, inhibiting CalcR-expressing NTS neurons increases food intake and partially prevents the appetite-suppressing effects of exendin-4, while their silencing leads to weight gain on a high-fat diet [61, 63]. These latter findings established CalcR-expressing NTS neurons (and their downstream targets) as physiological controls of energy balance. This concept was corroborated in a study on prolactin-releasing hormone (PRLH)–expressing NTS neurons, which represent a subset of CalcR-expressing NTS neurons [79]. Considering that the amylin receptor agonist cagrilintide [80] is in clinical development for the treatment of obesity [81], CalcR-expressing NTS neurons may thus be possible targets.

Given that genetically defined neurons in the NTS like those that express CCK and the CalcR suppress food intake via separate pathways, it is possible that their combined activation has an additive effect, which proved to be the case [82]. In the context of bariatric surgery, these neuronal populations are more highly activated after VSG compared to sham surgery in mice; however, their silencing has no impact on the reduced food intake or weight loss caused by the procedure [82]. These findings suggest that VSG does not only recruit CCK-expressing/CalcR-expressing NTS neurons to exert its inhibitory effects on energy balance. Additionally, these findings provide a salient example of how activation of specific populations of neurons by an intervention does not necessarily imply that these neurons drive changes in behavior associated with that intervention. Considering that RYGB leads to increased meal-induced activation of NE-expressing NTS neurons in mice [36], it would be interesting to determine the impact of their silencing or ablation on food intake and body weight, with preliminary data suggesting a modest and temporary effect [83].

## The pons

The pons lies in the middle of the brainstem sandwiched in between the medulla oblongata and midbrain and sits just anterior to the cerebellum. Because of its anatomical location, the pons is involved in various functions ranging from sensory processing to motor control. As mentioned above, the lPBN of the pons is heavily implicated in the aversive and non-aversive control of feeding via CGRP-expressing and non-CGRP-expressing neurons, respectively. Indeed, GLP-1-expressing NTS neurons project to the lPBN [84, 85], and administration of exendin-4 into this brain region, where it has direct excitatory effects [85], robustly reduces food intake [84–86] and increases the expression of CGRP [85]. Conversely, the administration of exendin-9 into the lPBN increases food intake [84, 85]. Given that the dorsal pons has only recently been sequenced at the single-cell level of resolution [87], this will allow the future functional characterization of other cell types in this brain region that control food intake and body weight. Considering that the pons transcriptome in humans [88] is similar to that of mice, especially in the PBN [87], this is likely to be of clinical relevance.

Locus coeruleus (LC) neurons of the pons are the major NE system of the brain and are traditionally implicated in arousal. Dating as far back as the 1930s, this system has been implicated in appetite suppression and weight loss due to its targeting by amphetamines [89], which has been confirmed by contemporary neuroscience techniques. For example, activating LC-NE neurons robustly suppresses food intake and causes weight loss in mice [90]. Curiously, activating LC-NE neurons causes hungry mice to drop their food, suggesting that these neurons serve to distract from feeding [90]. In the context of GLP-1R agonists, GLP-1-expressing NTS neurons project to the LC [69], and administration of exendin-4 into this brain region robustly suppresses food intake and causes an aversive response in rats [91]. In electrophysiology experiments, exendin-4 increases glutamatergic input onto LC-NE neurons [91]. Importantly, systemic semaglutide activates LC-NE neurons, and its acute appetite-suppressing effects are blunted when GLP-1Rs in the LC are blocked with local infusion of exendin-9 [91]. While it remains to be established if specifically inhibiting LC-NE neurons has a similar effect, these findings suggest that systemic GLP-1R agonists activate presynaptic GLP-1Rs on glutamatergic terminals synapsing onto LC-NE neurons, leading to their activation and suppression of food intake. When considering the findings of Sciolino et al. [90], the dependance of systemic semaglutide on LC GLP-1R activation in suppressing food intake might explain anecdotal evidence that people taking semaglutide often report no longer being preoccupied with thoughts about food.

The lateral dorsal tegmental nucleus (LDTg) of the pons receives dense axonal projections from the hindbrain. Systemically administered, fluorescently labelled exendin-4 accesses the LDTg, and administration of exendin-4 into this brain region suppresses food intake without causing aversion [92], unlike in the LC [91]. Conversely, administration of exendin-9 into the LDTg increases food intake [92]. Importantly, like the LC, the acute inhibitory effect of systemic exendin-4 on food intake is prevented when GLP-1Rs are blocked by exendin-9 in the LDTg [92]. Together, these findings suggest that systemic GLP-1R agonists suppress food intake via aversive and non-aversive pathways in the pons via GLP-1R-expressing LC and LDTg neurons, respectively.

## The midbrain

The midbrain lies in the uppermost part of the brainstem and is traditionally implicated in visual, auditory, and pain processing. Evidence suggests that the midbrain is also involved in the control of food intake. The midbrain dopaminergic system in particular has been heavily implicated in the rewarding or hedonic aspects of eating, especially the projection from the ventral tegmental area (VTA) to the ventral striatum (the mesolimbic pathway) [93]. For example, activating dopaminergic VTA neurons increases operant responses for a food reward [94], while inhibiting these neurons by activating GABAergic VTA interneurons has the opposite effect [95]. Further, sucrose infusion into the gut activates dopaminergic VTA neurons in a vagal afferent-dependent manner to reinforce its intake [96]. Conversely, activating GLP-1-expressing NTS neurons reduces high-fat diet intake by inhibiting dopaminergic VTA neurons through both reduced glutamatergic and enhanced GABAergic input [97].

In the context of GLP-1R agonists, GLP-1-expressing NTS neurons project to the VTA [98], and administration of exendin-4 into this brain region reduces food intake [98–100] and the motivation to obtain a food reward [99, 100] without causing aversion [98–100]. Moreover, the acute appetite-suppressing effects of systemic exendin-4 are diminished by the administration of a GLP-1R or AMPA receptor antagonist into the VTA [99]. In electrophysiological experiments, exendin-4 increases glutamatergic input onto dopaminergic VTA neurons via activation of presynaptic GLP-1Rs on glutamatergic afferents [99], analogous to the mechanism in the LC described above [91]. Additionally, co-administration of nicotine with liraglutide has additive inhibitory effects on food intake and body weight associated with increased activity of dopaminergic VTA neurons [101], while the GLP-1-MK-801 conjugate similarly has additive effects on food intake and body weight associated with increased activity of VTA neurons [60]. Clearly, future studies need to be performed to clarify the role of dopaminergic VTA neurons in controlling food intake and body weight. Interestingly, while GLP-1-expressing NTS neurons also project to the ventral striatum [102] and administration of exendin-4 into this brain region reduces food intake and the motivation to obtain a food reward [100, 102], this treatment does not affect ventral striatal dopamine release [103]. Instead, electrophysiological and behavioral experiments suggest that exendin-4 increases the activity of ventral striatal neurons and reduces food intake, respectively, via presynaptic GLP-1 receptors in glutamatergic neurons and subsequent postsynaptic AMPA receptor activation [103], analogous to the aforementioned mechanism in the VTA [99].

Another important dopaminergic population of neurons in the midbrain arises from the substantia nigra and projects to the dorsal striatum (the nigrostriatal pathway). While these neurons were traditionally implicated in controlling motor function, accumulating evidence suggests that they are also involved in food reward receipt, particularly in the form of fat as part of a gut-brain pathway. For example, dorsal striatal dopamine release from intragastric fat infusion is blunted in obesity associated with reduced levels of the fat-derived signaling molecule oleoylethanolamide (OEA) in the small intestine in mice [104]. Accordingly, supplementing OEA to obese mice reverses this state of dorsal striatal dopamine deficit in a vagal-afferent–dependent manner [104]. Moreover, the suppression of fat intake from systemic OEA is prevented when a mixed dopamine 1 receptor (D1R) and dopamine 2 receptor (D2R) antagonist is infused into the dorsal striatum [104]. In the context of bariatric surgery, RYGB increases small intestinal OEA synthesis associated with increases in dorsal striatal dopamine release and D1R availability in rats [105]. Moreover, the suppressed fat intake after RYGB is prevented by blocking intestinal OEA signaling, vagotomy, or blocking dorsal striatal D1R signaling [105]. This pathway was recently defined more precisely, indicating that right vagal afferents that innervate the duodenum form part of an elaborate polysynaptic circuit involving neurons in the right ventromedial NTS, non-CGRP-expressing neurons in the dorsal lPBN and dopaminergic neurons in the substantia nigra [106]. Further, the appetite-suppressing effects of CCK are blocked with ablation of right vagal afferents, neurons in the lPBN and substantia nigra, and antagonism of D1R/D2Rs in the dorsal striatum [106], suggesting overlapping central mechanisms of CCK with RYGB.

The midbrain is also home to serotonergic neurons of the dorsal raphe nucleus (DRN), which sends widespread ascending and descending projections throughout the brain. These neurons were traditionally implicated in regulating affect and are the target of selective serotonin reuptake inhibitors (SSRIs) like fluoxetine, which are clinically approved antidepressants. These drugs are also appetite suppressants and were prescribed for obesity until they were withdrawn owing to negative side effects [107]. Serotonin-expressing neurons of the DRN are also glutamatergic and are activated by feeding and PYY_3–36_, and their activation suppresses food intake [108]. Interestingly, an adjacent population of GABAergic neurons is activated by fasting but inhibited by the proopiomelanocortin (POMC) cleavage product alpha-melanocyte stimulating hormone (alpha-MSH), and their activation increases food intake [108]. In the context of GLP-1R agonists, GLP-1-expressing NTS neurons send projections to serotonergic DRN neurons, and chemical depletion of serotonin as well as blockade of central 5-HT_2A_ receptors, but not 5-HT_2C_ receptors, prevents the inhibitory effects of chronic central exendin-4 on food intake and body weight in rats [109]. Moreover, the inhibitory effects of chronic systemic liraglutide on food intake and body weight are blunted with blockade of central 5-HT_2A_ receptors [109]. These findings were unexpected as the 5-HT_2C_ receptor agonist lorcaserin was a previously used treatment for obesity prior to being recalled from increasing the risk of cancer development and as the appetite-suppressing effects of acute peripheral GLP-1 are diminished in 5-HT_2C_ receptor KO mice [110, 111]. However, the effects of acute peripheral liraglutide are preserved in 5-HT_2C_ receptor KO mice [111], again suggesting different central pathways are targeted by endogenous GLP-1 and GLP-1R agonists. Interestingly, RYGB controls 5-HT_2A_ receptor availability in the ventral striatum in rats [112], suggesting that GLP-1R agonists and bariatric surgery may both target the midbrain serotonergic system to exert their inhibitory effects on energy balance.

Neurons in the midbrain periaqueductal grey (PAG) surrounding the cerebral aqueduct have long been implicated in the modulation of pain as well as in the learning and action of defensive and aversive behaviors [113]. Accumulating evidence suggests that PAG neurons also control energy balance. For example, activation of GABAergic neurons in the ventrolateral (vlPAG) suppresses food intake, while their inhibition increases food intake [114]. Further, chronic activation of GABAergic vlPAG neurons causes marked weight loss in diet-induced obese mice due to both a suppression of food intake and an increase in energy expenditure [115]. These neurons reduce their activity before meal initiation, suggesting that they normally serve as a break to feeding [115]. Remarkably, this inhibitory effect is potentiated after chronic consumption of a high-fat diet due to a marked increase in GABAergic input and reduced expression of the calcium channel subunit Cacna2d1 [115]. Accordingly, viral-mediated restoration of Cacna2d1 in vlPAG GABAergic neurons normalizes GABAergic input in these neurons and markedly reduces body weight due to reductions in food intake and increases in energy expenditure [115]. These findings suggest that a high-fat diet impacts inhibitory plasticity in the midbrain, which can be corrected by restoring normal neuronal function. Notably, GLP-1-expressing NTS neurons project to the PAG [69] where GLP-1Rs are highly expressed [66], although it remains unclear what effects GLP-1R agonists have on energy balance in this brain region.

## The hypothalamus

The hypothalamus situated at the base of forebrain has long been implicated in body weight control [116, 117]. Key regions of the hypothalamus in this regard include the ventromedial hypothalamus (VMH), dorsomedial hypothalamus (DMH), PVH, and the lateral hypothalamic area (LHA), which all receive projections from GLP-1-expressing NTS neurons, where deletion or blockade of GLP-1Rs increases food intake and body weight [72, 118–120]. The arcuate nucleus (ARC) at the floor of the third ventricle has received particular attention because it lies either side of the median eminence (ME) which, like the AP, is not fully shielded by the blood–brain barrier [121] and has fenestrated capillaries therefore permitting access to circulating factors that control energy balance [122]. In the case of the fat-derived hormone leptin, this is believed to be facilitated by tanycytes lining the floor of the third ventricle in a two-step process. First, leptin is taken up by tanycytic end feet adjacent to ME capillaries and is transported into the CSF of the third ventricle in a leptin receptor-dependent manner [123, 124]. Second, leptin is transported back into the brain via tanycytes that protrude to hypothalamic nuclei [123, 124].

A similar uptake mechanism has been proposed for liraglutide based on evidence showing that inhibiting ME tanycytic function prevents activation of neurons in the PVH, DMH, and LHA as well as food intake suppression and weight loss from chronic systemic liraglutide treatment [125]. Further, deleting GLP-1Rs in ME tanycytes has similar effects [125], suggesting that liraglutide first activates GLP-1Rs in ME tanycytes to then gain access to and activate GLP-1R-expressing hypothalamic neurons and exert its effects on energy balance. Indeed, systemically administered, fluorescently labeled liraglutide and semaglutide gain access to the median eminence and ARC and to a lesser extent the PVH and DMH [51, 126], while administration of liraglutide into the ARC, PVH, and LHA (but not the DMH and VMH) robustly reduces food intake [120, 127]. Conversely, the combined deletion of GLP-1Rs in hypothalamic neurons (ARC, VMH, and DMH) partially prevents the weight-lowering effects of chronic systemic liraglutide treatment [128, 129]. On the other hand, ablating the PVH or deleting GLP-1Rs in this brain region does not prevent the inhibitory effects on food intake and body weight of chronic systemic liraglutide [126, 130] or exendin-4 [128, 131]. Similarly, deleting GLP-1Rs in the VMH does not prevent the inhibitory effects of chronic systemic exendin-4 and liraglutide on food intake and body weight [132]. Taken together, these data suggest that GLP-1R agonists need to engage multiple extrahypothalamic and hypothalamic populations simultaneously to exert their inhibitory effects on food intake and body weight, at least in the chronic setting.

Within the ARC, two functionally opposing populations of neurons exist which co-express agouti-related peptide (AgRP) and neuropeptide Y (NPY) or POMC [133]. Acute studies generally show that activating ARC AgRP/NPY neurons increases food intake, while activating ARC POMC neurons suppresses food intake with the opposite profile seen with their respective inhibition [134–138]. Indeed, liraglutide is thought to acutely suppress food intake by activating GABAergic GLP-1R-expressing DMH neurons which project to and inhibit ARC AgRP/NPY neurons as determined by patch-clamp electrophysiology experiments on hypothalamic slices [129]. However, chronic inhibition or ablation of ARC AgRP/NPY neurons or chronic activation of ARC POMC neurons fails to affect body weight in a stable manner [139–142]. These findings have implications for obesity drug development and suggest that strategies need to be developed that not only target ARC AgRP/NPY or POMC neurons. Notably, simultaneously activating ARC AgRP/NPY neurons and inhibiting POMC neurons additively increases food intake in the acute setting [143]. It would therefore be interesting to determine if the opposite approach, i.e., simultaneously inhibiting ARC AgRP/NPY neurons and activating ARC POMC neurons, additively reduces food intake and leads to weight loss in the chronic setting as this would be therapeutically relevant. Another consideration to make when using this approach is the potential aversive effects of manipulating ARC AgRP/NPY neuronal activity. While activating ARC AgRP/NPY neurons has been shown to be both aversive [136] and rewarding [144] depending on the duration of activation, the positive valence associated with inhibiting ARC AgRP/NPY neurons [136] lends itself well to targeting these neurons for obesity drug development.

Like the other neuronal types discussed in this Review, the function of ARC AgRP/NPY and ARC POMC neurons has been interrogated in real-time in vivo in response to different treatments using fiber photometry. This revealed that ghrelin robustly activates ARC AgRP/NPY neurons as soon as 30 s after its systemic administration, while it robustly inhibits ARC POMC neurons after a 15-min delay suggesting an indirect effect [145]. These results are entirely consistent with the known orexigenic effects of this stomach-derived hormone [146, 147]. In contrast, intragastric infusion of lipids, glucose, and amino acids all robustly suppresses the activity of ARC AgRP/NPY neurons as does systemic administration of CCK and PYY_3–36_ [148, 149]. Further, the suppression of ARC AgRP/NPY neurons by intragastric lipids is blocked by a CCK receptor antagonist [148] and is mediated by vagal afferents, while that of glucose is mediated by spinal afferents [150]. Notably, fiber photometry also revealed that systemic administration of GLP-1 [149] and liraglutide [148] does not affect the activity of ARC AgRP/NPY neurons, emphasizing how findings from patch-clamp electrophysiology experiments on hypothalamic slices can differ from the situation in vivo. Taken together [150], these findings suggest that nutrients and gut hormones may exert direct and indirect effects on the activity of ARC AgRP/NPY neurons via separate ascending neural pathways.

Single-cell RNA sequencing has revealed many cell types in the ARC [151, 152]. This analysis specifically in ARC POMC neurons revealed that leptin receptor-expressing and GLP-1R-expressing neurons are non-overlapping [153], which was confirmed in an independent study using in situ hybridization and transgenic reporter mice [154]. Acutely activating GLP-1R-expressing ARC POMC neurons robustly suppresses food intake [154]. Accordingly, acute administration of exendin-4 (but not GLP-1) into the ARC potently reduces food intake [128], while liraglutide directly activates ARC POMC neurons [126, 155] and its weight-lowering effects upon chronic systemic administration are partially prevented when exendin-9 is co-administered into the ARC [126]. On the other hand, the appetite-suppressing effects of chronic systemic exendin-4 are preserved in mice lacking GLP-1Rs in ARC POMC neurons [128] or when ARC neurons are ablated [34]. Similarly, the appetite-suppressing and weight-lowering effects of chronic systemic liraglutide are preserved in mice lacking GLP-1Rs in ARC neurons [130]. Collectively, the genetic findings suggest that targeting ARC neurons is not necessary for the action of GLP-1R agonists. This is underscored by the finding that ablating GABAergic basonuclin 2 (BNC2)-expressing neurons in the ARC, which strongly inhibit ARC AgRP neurons to mediate the effects of leptin on energy balance, has no impact on the acute appetite suppressing effects of semaglutide [156]. In the context of bariatric surgery, rats lacking melanocortin 4 receptors (MC4Rs), which are activated by the POMC-cleavage product alpha-MSH [157], are still responsive to VSG [158]; on the other hand, mice lacking MC4Rs are less responsive to RYGB [159], suggesting that ARC POMC neurons are targeted by this particular bariatric surgery. Since MC4R-mediated suppression of appetite is at the level of PVH neurons projecting to the non-CGRP-expressing neurons in the lPBN [160], these findings suggest that RYGB targets this pathway.

## The zona incerta

The ZI is a horizontally elongated gray matter region situated in between the hypothalamus and thalamus and is implicated in a broad array of functions ranging from sleep to predator avoidance [161]. Accumulating evidence suggests that ZI neurons control energy balance [161]. For example, GABAergic neurons of the ZI are activated by ghrelin and their activation robustly increases the intake of high-fat food by inhibiting glutamatergic neurons of the PVT [162]. Notably, a glutamatergic projection from the parasubthalamic nucleus (PSN) activates the same glutamatergic PVT neurons to suppress food intake [162]. Considering that ileal GLP-1 activates glutamatergic PSN neurons to suppress food intake [39], glutamatergic PVT neurons might be the next order neuron in this intricate pathway. Interestingly, semaglutide robustly activates PSN and PVT neurons, possibly by tanycyte-mediated uptake [51], suggesting a convergence of endogenous GLP-1 and GLP-1R agonist action in the neural pathways that suppress food intake in the PVT. In line with this idea, inhibiting tanycyte function prevents the activation of neurons in the ZI by liraglutide [125]. Since the GLP-1R is highly expressed in the upstream PSN and ZI [66], it would be interesting to determine their neurochemical phenotype and whether they are activated by peripheral GLP-1R agonists to suppress food intake. Along these lines, GLP-1-expressing NTS neurons project to the PVT, and administration of exendin-4 into this brain region reduces food intake and motivation for food reward, while exendin-9 has the opposite effect [163]. These PVT neurons in turn project to the ventral striatum and electrophysiological experiments revealed that GLP-1R activation in the PVT reduces excitatory drive in this region [163].

Dopaminergic neurons are also found in the ZI and have similar effects on feeding as GABAergic ZI neurons [164]. Remarkably, while dopaminergic VTA neurons have been extensively studied in the context of food reward and obesity, their ablation has little overall effect on food intake and body weight [164]. In striking contrast, ablating dopaminergic ZI neurons reduces food intake and weight gain associated with reduced motivation to obtain a food reward [164], while activating dopaminergic ZI neurons robustly increases food intake and motivation to obtain a food reward [164]. Interestingly, chronic consumption of a high-fat high-sugar (HFHS) diet depolarizes resting membrane potential and increases excitatory input onto dopaminergic ZI neurons [164]. Similar to GABAergic ZI neurons, these dopaminergic ZI neurons project to and inhibit PVT neurons to robustly increase food intake [164]. These findings provide strong evidence that dopaminergic ZI neurons contribute to hedonic feeding and obesity, perhaps even more so than dopaminergic VTA neurons making them interesting candidates to study in the context of both bariatric surgery and GLP-1R agonists.

## The septum

The septum is a subcortical forebrain area that controls various processes ranging from sleep to learning and memory. GLP-1-expressing NTS neurons project to the lateral septum, and administration of exendin-4 into this brain region suppresses food intake [165]. Conversely, administration of exendin-9 into the lateral septum increases food intake, prevents the ability of nutrient load to suppress food intake [165], prevents stress-induced hypophagia [166], and increases the motivation to obtain a sucrose reward [167]. Accordingly, chronic activation of GLP-1R-expressing lateral septum neurons reduces food intake and body weight as well as causing aversion [130] while acutely inhibiting GLP-1R-expressing lateral septal neurons robustly increases food intake [168], and their silencing leads to weight gain on a high-fat diet [130]. At the circuit level, GLP-1R-expressing lateral septum neurons make GABAergic connections with the LHA where activation of their terminals decreases food intake [168]. Accordingly, exendin-4 in the lateral septum enhances GABA release onto LHA neurons [168]. Importantly, systemic liraglutide activates GLP-1R-expressing lateral septum neurons, while their silencing or deletion of GLP-1Rs partially attenuates the inhibitory effects of chronic systemic liraglutide on food intake and body weight in mice [130].

## The *hippocampus*

**Fig. 2:**
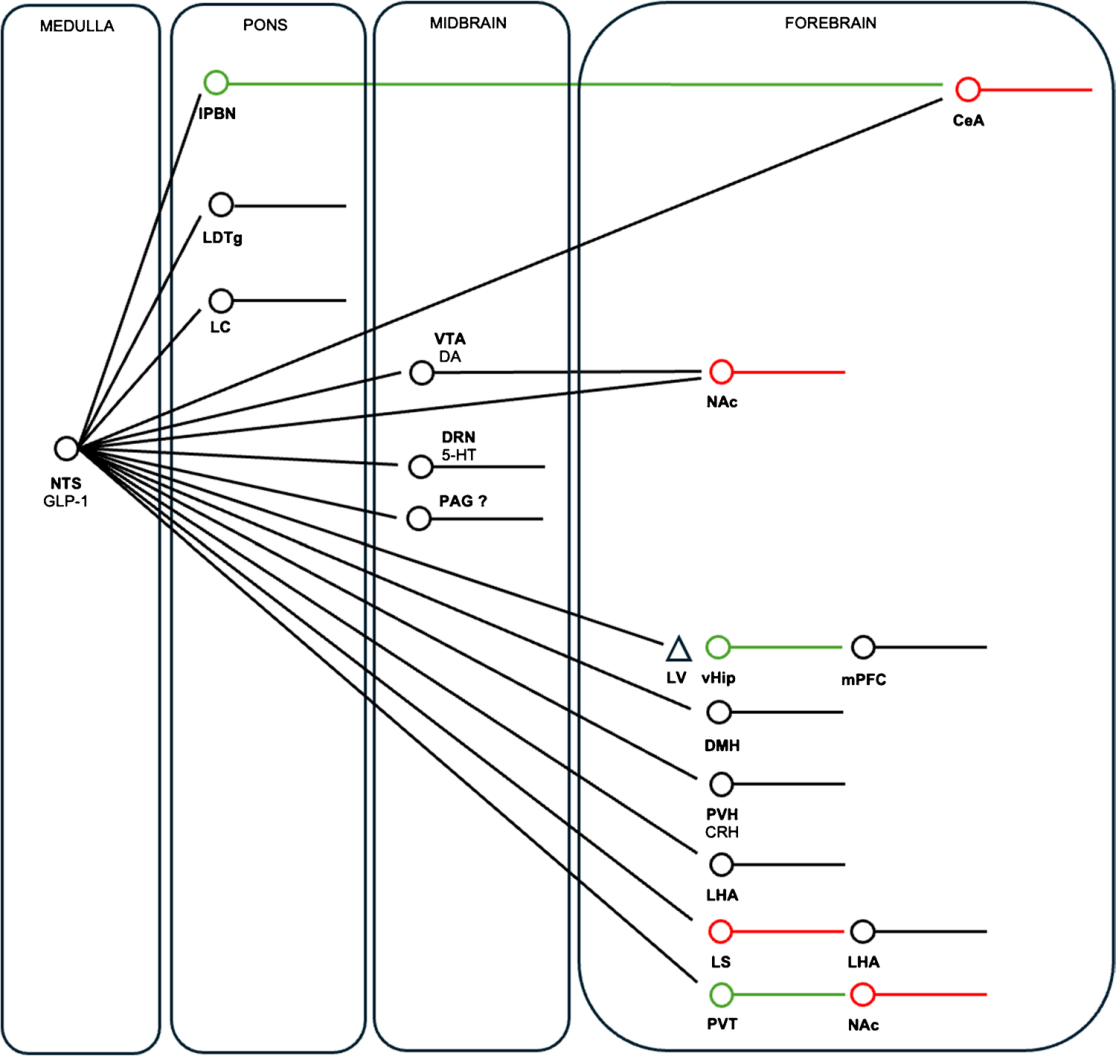
Wiring diagram for central GLP-1 projections and sites of appetite suppression and weight loss. This schematic summarizes the central projections of GLP-1-expressing NTS neurons and where local administration of GLP-1R agonists and antagonists control food intake or body weight. In the VTA and PVH, GLP-1 has presynaptic effects on glutamatergic and/or GABAergic neurons. Neurons in red are GABAergic, and neurons in green are glutamatergic

The hippocampus is traditionally implicated in learning and memory. Accumulating evidence suggests that the ventral hippocampus contributes to the control of energy balance. For example, administration of leptin into the ventral hippocampus decreases food intake [169], while that of ghrelin increases food intake [170]. In the context of GLP-1R agonists, administration of exendin-4 into the ventral hippocampus reduces food intake without causing malaise, while exendin-9 increases food intake [171]. Further work showed that GLP-1R-expressing ventral hippocampal neurons are monosynaptically connected to the medial prefrontal cortex (mPFC), and the suppression of food intake and motivated behaviors by intra-ventral hippocampal administration of exendin-4 is lost with their inhibition or antagonism of NMDARs [172]. However, it remains unclear if this pathway is targeted by systemic GLP-1R agonist treatment (Fig. 2).

## Summary

**Fig. 3:**
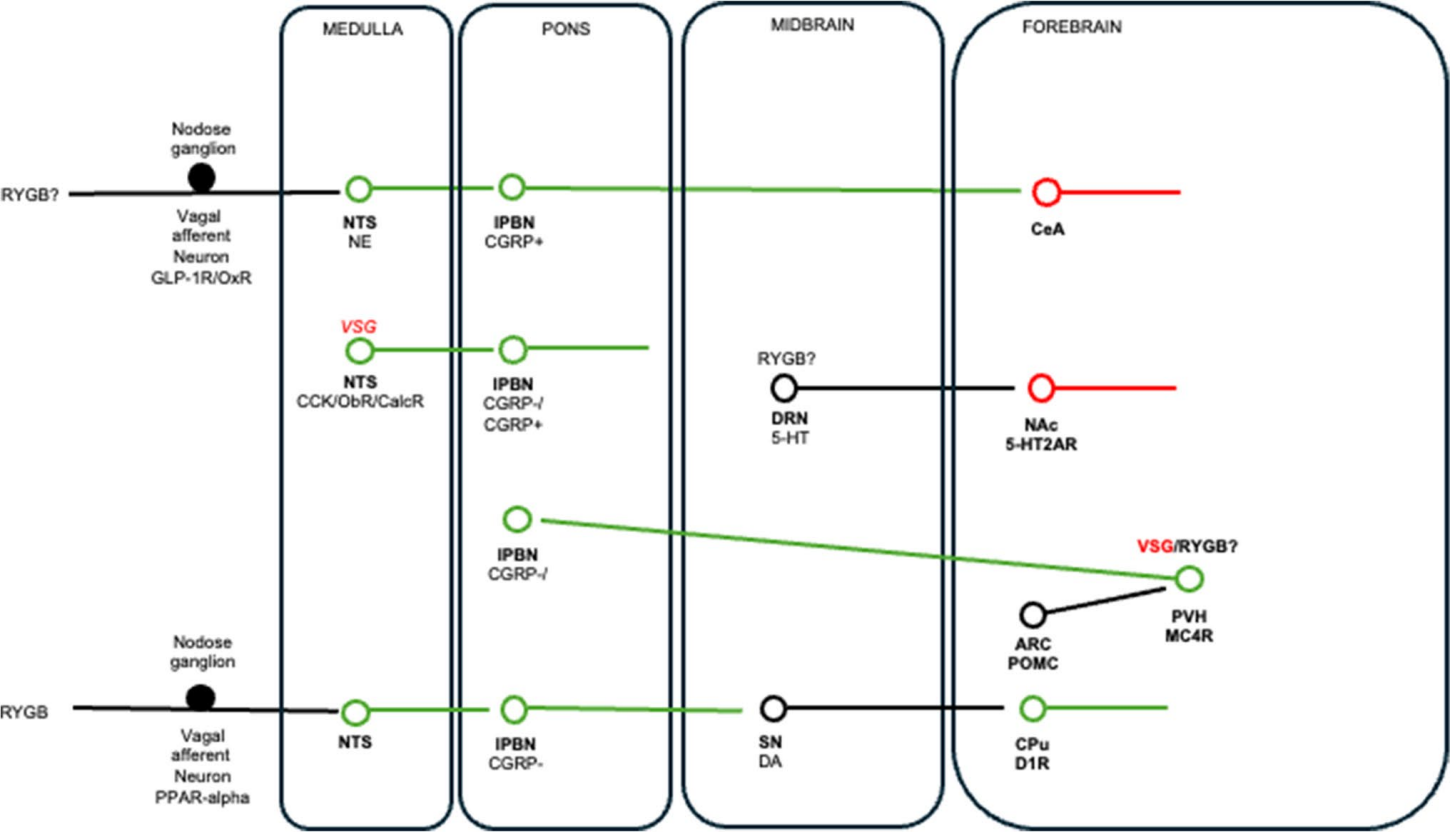
Wiring diagram for bariatric surgery-mediated appetite suppression and weight loss. This schematic summarizes peripheral and central neural pathways targeted by RYGB and VSG. Text in red italics highlights regions where VSG does not exert its effects based on neuronal inhibition approaches. Neurons in red are GABAergic, and neurons in green are glutamatergic

In this Review, we have summarized the peripheral and central neural pathways targeted by endogenous GLP-1 and GLP-1R agonists (Fig. 1). Owing to the development of sophisticated neuroscientific techniques for assessing neuronal activity and connectivity, tremendous progress has been made in defining these neural pathways at an unprecedented level of molecular, anatomical, functional, and behavioral detail. Overall, evidence suggests that GLP-1R agonists directly and indirectly target a distributed network of neurons to exert their effects on food intake and body weight (Fig. 1). Much less is known about the neural circuits targeted by bariatric surgery (Fig. 3), and there is a pressing need to apply the methodology used for GLP-1R agonists in the corresponding rodent models of RYGB and VSG in this context [173]. This is of potential clinical relevance, as further defining the commonalities and differences between GLP-1R agonists and bariatric surgery in terms of their central mechanism of action may not only help bridge the gap between the pharmacological and surgical treatments for obesity but may also provide a neural basis for their combined use when each individually fail [174, 175].

## Data Availability

No datasets were generated or analysed during the current study.

## References

[1] Haslam DW, James WP (2005) Obesity. Lancet 366(9492):1197–1209 16198769 10.1016/S0140-6736(05)67483-1

[2] Collaborators GBDO et al (2017) Health effects of overweight and obesity in 195 countries over 25 years. N Engl J Med 377(1):13–27 28604169 10.1056/NEJMoa1614362 PMC5477817

[3] Wilding JPH et al (2021) Once-weekly semaglutide in adults with overweight or obesity. N Engl J Med 384(11):989–1002 33567185 10.1056/NEJMoa2032183

[4] Frias JP et al (2021) Tirzepatide versus semaglutide once weekly in patients with type 2 diabetes. N Engl J Med 385(6):503–515 34170647 10.1056/NEJMoa2107519

[5] Jastreboff AM et al (2023) Triple-hormone-receptor agonist retatrutide for obesity - a phase 2 trial. N Engl J Med 389(6):514–526 37366315 10.1056/NEJMoa2301972

[6] Muller TD et al (2022) Anti-obesity drug discovery: advances and challenges. Nat Rev Drug Discov 21(3):201–223 34815532 10.1038/s41573-021-00337-8 PMC8609996

[7] Kanoski SE et al (2011) Peripheral and central GLP-1 receptor populations mediate the anorectic effects of peripherally administered GLP-1 receptor agonists, liraglutide and exendin-4. Endocrinology 152(8):3103–3112 21693680 10.1210/en.2011-0174 PMC3138234

[8] Sisley S et al (2014) Neuronal GLP1R mediates liraglutide’s anorectic but not glucose-lowering effect. J Clin Invest 124(6):2456–2463 24762441 10.1172/JCI72434 PMC4038572

[9] Dicker D et al (2024) Bariatric metabolic surgery vs glucagon-like peptide-1 receptor agonists and mortality. JAMA Netw Open 7(6):e2415392 38848064 10.1001/jamanetworkopen.2024.15392 PMC11161844

[10] Chambers AP et al (2014) Regulation of gastric emptying rate and its role in nutrient-induced GLP-1 secretion in rats after vertical sleeve gastrectomy. Am J Physiol Endocrinol Metab 306(4):E424–E432 24368666 10.1152/ajpendo.00469.2013 PMC3923088

[11] Chaudhari SN et al (2021) Bariatric surgery reveals a gut-restricted TGR5 agonist with anti-diabetic effects. Nat Chem Biol 17(1):20–29 32747812 10.1038/s41589-020-0604-z PMC7891870

[12] Larraufie P et al (2019) Important role of the GLP-1 axis for glucose homeostasis after bariatric surgery*.* Cell Rep 26(6):1399–1408 10.1016/j.celrep.2019.01.047 PMC6367566 30726726

[13] Zhai H et al (2018) Takeda G protein-coupled receptor 5-mechanistic target of rapamycin complex 1 signaling contributes to the increment of glucagon-like peptide-1 production after Roux-en-Y gastric bypass. EBioMedicine 32:201–214 29859856 10.1016/j.ebiom.2018.05.026 PMC6020750

[14] Papamargaritis D, le Roux CW (2021) Do gut hormones contribute to weight loss and glycaemic outcomes after bariatric surgery? Nutr 13(3) 10.3390/nu13030762 PMC7996890 33652862

[15] Mokadem M et al (2014) Effects of Roux-en-Y gastric bypass on energy and glucose homeostasis are preserved in two mouse models of functional glucagon-like peptide-1 deficiency. Mol Metab 3(2):191–201 24634822 10.1016/j.molmet.2013.11.010 PMC3953682

[16] Wilson-Perez HE et al (2013) Vertical sleeve gastrectomy is effective in two genetic mouse models of glucagon-like peptide 1 receptor deficiency. Diabetes 62(7):2380–2385 23434938 10.2337/db12-1498 PMC3712071

[17] Ye J et al (2014) GLP-1 receptor signaling is not required for reduced body weight after RYGB in rodents. Am J Physiol Regul Integr Comp Physiol 306(5):R352–R362 24430883 10.1152/ajpregu.00491.2013 PMC3949077

[18] Carmody JS et al (2016) Peripheral, but not central, GLP-1 receptor signaling is required for improvement in glucose tolerance after Roux-en-Y gastric bypass in mice. Am J Physiol Endocrinol Metab 310(10):E855–E861 27026085 10.1152/ajpendo.00412.2015 PMC4888530

[19] Osto E et al (2015) Rapid and body weight-independent improvement of endothelial and high-density lipoprotein function after Roux-en-Y gastric bypass: role of glucagon-like peptide-1. Circulation 131(10):871–881 25673670 10.1161/CIRCULATIONAHA.114.011791

[20] Buller S, Blouet C (2024) Brain access of incretins and incretin receptor agonists to their central targets relevant for appetite suppression and weight loss. Am J Physiol Endocrinol Metab 326(4):E472–E480 38381398 10.1152/ajpendo.00250.2023 PMC11193531

[21] Williams DL, Baskin DG, Schwartz MW (2009) Evidence that intestinal glucagon-like peptide-1 plays a physiological role in satiety. Endocrinology 150(4):1680–1687 19074583 10.1210/en.2008-1045 PMC2659282

[22] Neuhuber WL, Berthoud HR (2021) Functional anatomy of the vagus system - emphasis on the somato-visceral interface. Auton Neurosci 236:102887 34634680 10.1016/j.autneu.2021.102887 PMC8627476

[23] Date Y et al (2002) The role of the gastric afferent vagal nerve in ghrelin-induced feeding and growth hormone secretion in rats. Gastroenterology 123(4):1120–1128 12360474 10.1053/gast.2002.35954

[24] Broberger C et al (2001) Expression and regulation of cholecystokinin and cholecystokinin receptors in rat nodose and dorsal root ganglia. Brain Res 903(1–2):128–140 11382396 10.1016/s0006-8993(01)02468-4

[25] Smith GP et al (1981) Abdominal vagotomy blocks the satiety effect of cholecystokinin in the rat. Science 213(4511):1036–1037 7268408 10.1126/science.7268408

[26] Nakagawa A et al (2004) Receptor gene expression of glucagon-like peptide-1, but not glucose-dependent insulinotropic polypeptide, in rat nodose ganglion cells. Auton Neurosci 110(1):36–43 14766323 10.1016/j.autneu.2003.11.001

[27] Koda S et al (2005) The role of the vagal nerve in peripheral PYY3-36-induced feeding reduction in rats. Endocrinology 146(5):2369–2375 15718279 10.1210/en.2004-1266

[28] Krieger JP et al (2016) Knockdown of GLP-1 receptors in vagal afferents affects normal food intake and glycemia. Diabetes 65(1):34–43 26470787 10.2337/db15-0973

[29] Alonso AM et al (2024) The vagus nerve mediates the physiological but not pharmacological effects of PYY(3–36) on food intake. Mol Metab 81:101895 38340808 10.1016/j.molmet.2024.101895 PMC10877939

[30] Mirabella PN, Fenselau H (2023) Advanced neurobiological tools to interrogate metabolism. Nat Rev Endocrinol 19(11):639–654 37674015 10.1038/s41574-023-00885-6

[31] Bai L et al (2019) Genetic identification of vagal sensory neurons that control feeding. Cell 179(5):1129–1143 31730854 10.1016/j.cell.2019.10.031 PMC6916730

[32] Williams EK et al (2016) Sensory neurons that detect stretch and nutrients in the digestive system. Cell 166(1):209–221 27238020 10.1016/j.cell.2016.05.011 PMC4930427

[33] Munzberg H, Berthoud HR, Neuhuber WL (2023) Sensory spinal interoceptive pathways and energy balance regulation. Mol Metab 78:101817 37806487 10.1016/j.molmet.2023.101817 PMC10590858

[34] Huang KP et al (2024) Dissociable hindbrain GLP1R circuits for satiety and aversion. Nat 10.1038/s41586-024-07685-6 PMC12519567 38987598

[35] Varin EM et al (2019) Distinct neural sites of GLP-1R expression mediate physiological versus pharmacological control of incretin action. Cell Rep 27(11):3371–3384 31189118 10.1016/j.celrep.2019.05.055

[36] Mumphrey MB et al (2016) Eating in mice with gastric bypass surgery causes exaggerated activation of brainstem anorexia circuit. Int J Obes (Lond) 40(6):921–928 26984418 10.1038/ijo.2016.38 PMC4899289

[37] Borgmann D et al (2021) Gut-brain communication by distinct sensory neurons differently controls feeding and glucose metabolism. Cell Metab 33(7):1466–1482 34043943 10.1016/j.cmet.2021.05.002 PMC8280952

[38] Hao Z et al (2014) Vagal innervation of intestine contributes to weight loss after Roux-en-Y gastric bypass surgery in rats. Obes Surg 24(12):2145–2151 24972684 10.1007/s11695-014-1338-3 PMC4224982

[39] Zhang T et al (2022) An inter-organ neural circuit for appetite suppression. Cell 185(14):2478–2494 35662413 10.1016/j.cell.2022.05.007 PMC9433108

[40] Goldspink DA et al (2020) Labeling and characterization of human GLP-1-secreting L-cells in primary ileal organoid culture. Cell Rep 31(13):107833 32610134 10.1016/j.celrep.2020.107833 PMC7342002

[41] Reimann F et al (2008) Glucose sensing in L cells: a primary cell study. Cell Metab 8(6):532–539 19041768 10.1016/j.cmet.2008.11.002 PMC2697331

[42] Bai L et al (2022) Enteroendocrine cell types that drive food reward and aversion. Elife 11 10.7554/eLife.74964 PMC9363118 35913117

[43] Kaelberer MM et al (2018) A gut-brain neural circuit for nutrient sensory transduction. Sci 361(6408) 10.1126/science.aat5236 PMC6417812 30237325

[44] Cai Y, Hay M, Bishop VS (1996) Synaptic connections and interactions between area postrema and nucleus tractus solitarius. Brain Res 724(1):121–124 8816265 10.1016/0006-8993(96)00282-x

[45] Shapiro RE, Miselis RR (1985) The central neural connections of the area postrema of the rat. J Comp Neurol 234(3):344–364 3988989 10.1002/cne.902340306

[46] Maolood N, Meister B (2009) Protein components of the blood-brain barrier (BBB) in the brainstem area postrema-nucleus tractus solitarius region. J Chem Neuroanat 37(3):182–195 19146948 10.1016/j.jchemneu.2008.12.007

[47] Merchenthaler I, Lane M, Shughrue P (1999) Distribution of pre-pro-glucagon and glucagon-like peptide-1 receptor messenger RNAs in the rat central nervous system. J Comp Neurol 403(2):261–280 9886047 10.1002/(sici)1096-9861(19990111)403:2<261::aid-cne8>3.0.co;2-5

[48] Kieffer TJ, McIntosh CH, Pederson RA (1995) Degradation of glucose-dependent insulinotropic polypeptide and truncated glucagon-like peptide 1 in vitro and in vivo by dipeptidyl peptidase IV. Endocrinology 136(8):3585–3596 7628397 10.1210/endo.136.8.7628397

[49] Lau J et al (2015) Discovery of the once-weekly glucagon-like peptide-1 (GLP-1) analogue semaglutide. J Med Chem 58(18):7370–7380 26308095 10.1021/acs.jmedchem.5b00726

[50] Agerso H et al (2002) The pharmacokinetics, pharmacodynamics, safety and tolerability of NN2211, a new long-acting GLP-1 derivative, in healthy men. Diabetologia 45(2):195–202 11935150 10.1007/s00125-001-0719-z

[51] Gabery S et al (2020) Semaglutide lowers body weight in rodents via distributed neural pathways. JCI Insight 5(6) 10.1172/jci.insight.133429 PMC7213778 32213703

[52] Salinas CBG et al (2018) Integrated brain atlas for unbiased mapping of nervous system effects following liraglutide treatment. Sci Rep 8(1):10310 29985439 10.1038/s41598-018-28496-6 PMC6037685

[53] Alhadeff AL, Grill HJ (2014) Hindbrain nucleus tractus solitarius glucagon-like peptide-1 receptor signaling reduces appetitive and motivational aspects of feeding. Am J Physiol Regul Integr Comp Physiol 307(4):R465–R470 24944243 10.1152/ajpregu.00179.2014 PMC4137150

[54] Fortin SM et al (2020) GABA neurons in the nucleus tractus solitarius express GLP-1 receptors and mediate anorectic effects of liraglutide in rats. Sci Transl Med 12(533) 10.1126/scitranslmed.aay8071 PMC7211411 32132220

[55] Adams JM et al (2018) Liraglutide modulates appetite and body weight through glucagon-like peptide 1 receptor-expressing glutamatergic neurons. Diabetes 67(8):1538–1548 29776968 10.2337/db17-1385 PMC6054439

[56] Reiner DJ et al (2016) Astrocytes regulate GLP-1 receptor-mediated effects on energy balance. J Neurosci 36(12):3531–3540 27013681 10.1523/JNEUROSCI.3579-15.2016 PMC4804010

[57] Astrup A et al (2009) Effects of liraglutide in the treatment of obesity: a randomised, double-blind, placebo-controlled study. Lancet 374(9701):1606–1616 19853906 10.1016/S0140-6736(09)61375-1

[58] Zhang C et al (2021) Area postrema cell types that mediate nausea-associated behaviors. Neuron 109(3):461–472 10.1016/j.neuron.2020.11.010 PMC7864887 33278342

[59] Baraboi ED et al (2010) Lesions of area postrema and subfornical organ alter exendin-4-induced brain activation without preventing the hypophagic effect of the GLP-1 receptor agonist. Am J Physiol Regul Integr Comp Physiol 298(4):R1098–R1110 20106992 10.1152/ajpregu.00326.2009

[60] Petersen J et al (2024) GLP-1-directed NMDA receptor antagonism for obesity treatment. Nat 10.1038/s41586-024-07419-8 PMC11136670 38750368

[61] Roman CW, Derkach VA, Palmiter RD (2016) Genetically and functionally defined NTS to PBN brain circuits mediating anorexia. Nat Commun 7:11905 27301688 10.1038/ncomms11905 PMC4912612

[62] Cai H et al (2014) Central amygdala PKC-delta(+) neurons mediate the influence of multiple anorexigenic signals. Nat Neurosci 17(9):1240–1248 25064852 10.1038/nn.3767 PMC4146747

[63] Campos CA et al (2016) Parabrachial CGRP neurons control meal termination. Cell Metab 23(5):811–820 27166945 10.1016/j.cmet.2016.04.006 PMC4867080

[64] Roman CW, Sloat SR, Palmiter RD (2017) A tale of two circuits: CCK(NTS) neuron stimulation controls appetite and induces opposing motivational states by projections to distinct brain regions. Neuroscience 358:316–324 28684275 10.1016/j.neuroscience.2017.06.049 PMC5875425

[65] Carter ME, Han S, Palmiter RD (2015) Parabrachial calcitonin gene-related peptide neurons mediate conditioned taste aversion. J Neurosci 35(11):4582–4586 25788675 10.1523/JNEUROSCI.3729-14.2015 PMC4363386

[66] Cork SC et al (2015) Distribution and characterisation of glucagon-like peptide-1 receptor expressing cells in the mouse brain. Mol Metab 4(10):718–731 26500843 10.1016/j.molmet.2015.07.008 PMC4588458

[67] Ludwig MQ et al (2021) A genetic map of the mouse dorsal vagal complex and its role in obesity. Nat Metab 3(4):530–545 33767443 10.1038/s42255-021-00363-1 PMC12009600

[68] Gaykema RP et al (2017) Activation of murine pre-proglucagon-producing neurons reduces food intake and body weight. J Clin Invest 127(3):1031–1045 28218622 10.1172/JCI81335 PMC5330725

[69] Llewellyn-Smith IJ et al (2011) Preproglucagon neurons project widely to autonomic control areas in the mouse brain. Neuroscience 180:111–121 21329743 10.1016/j.neuroscience.2011.02.023 PMC4298012

[70] Williams DL et al (2018) GLP-1 action in the mouse bed nucleus of the stria terminalis. Neuropharmacology 131:83–95 29221794 10.1016/j.neuropharm.2017.12.007 PMC5840513

[71] Holt MK et al (2019) Preproglucagon neurons in the nucleus of the solitary tract are the main source of brain GLP-1, mediate stress-induced hypophagia, and limit unusually large intakes of food. Diabetes 68(1):21–33 30279161 10.2337/db18-0729 PMC6314470

[72] Liu J et al (2017) Enhanced AMPA receptor trafficking mediates the anorexigenic effect of endogenous glucagon-like peptide-1 in the paraventricular hypothalamus. Neuron 96(4):897–909 29056294 10.1016/j.neuron.2017.09.042 PMC5729931

[73] Scrocchi LA et al (1996) Glucose intolerance but normal satiety in mice with a null mutation in the glucagon-like peptide 1 receptor gene. Nat Med 2(11):1254–1258 8898756 10.1038/nm1196-1254

[74] Brierley DI et al (2021) Central and peripheral GLP-1 systems independently suppress eating. Nat Metab 3(2):258–273 33589843 10.1038/s42255-021-00344-4 PMC7116821

[75] Card JP et al (2018) GLP-1 neurons form a local synaptic circuit within the rodent nucleus of the solitary tract. J Comp Neurol 526(14):2149–2164 30019398 10.1002/cne.24482 PMC6193818

[76] Hisadome K et al (2010) Leptin directly depolarizes preproglucagon neurons in the nucleus tractus solitarius: electrical properties of glucagon-like peptide 1 neurons. Diabetes 59(8):1890–1898 20522593 10.2337/db10-0128 PMC2911066

[77] Dowsett GKC et al (2021) A survey of the mouse hindbrain in the fed and fasted states using single-nucleus RNA sequencing. Mol Metab 53:101240 33962048 10.1016/j.molmet.2021.101240 PMC8170503

[78] Cheng W et al (2020) Calcitonin receptor neurons in the mouse nucleus tractus solitarius control energy balance via the non-aversive suppression of feeding. Cell Metab 31(2):301–312 31955990 10.1016/j.cmet.2019.12.012 PMC7104375

[79] Cheng W et al (2021) NTS Prlh overcomes orexigenic stimuli and ameliorates dietary and genetic forms of obesity. Nat Commun 12(1):5175 34462445 10.1038/s41467-021-25525-3 PMC8405610

[80] Kruse T et al (2021) Development of cagrilintide, a long-acting amylin analogue. J Med Chem 64(15):11183–11194 34288673 10.1021/acs.jmedchem.1c00565

[81] Lau DCW et al (2021) Once-weekly cagrilintide for weight management in people with overweight and obesity: a multicentre, randomised, double-blind, placebo-controlled and active-controlled, dose-finding phase 2 trial. Lancet 398(10317):2160–2172 34798060 10.1016/S0140-6736(21)01751-7

[82] Qiu W et al (2023) Multiple NTS neuron populations cumulatively suppress food intake. Elife 12 10.7554/eLife.85640 PMC10781422 38059498

[83] Albaugh VL et al (2023) Regulation of body weight: lessons learned from bariatric surgery. Mol Metab 68:101517 35644477 10.1016/j.molmet.2022.101517 PMC9938317

[84] Alhadeff AL et al (2014) Glucagon-like peptide-1 receptor signaling in the lateral parabrachial nucleus contributes to the control of food intake and motivation to feed. Neuropsychopharmacology 39(9):2233–2243 24681814 10.1038/npp.2014.74 PMC4104342

[85] Richard JE et al (2014) GLP-1 receptor stimulation of the lateral parabrachial nucleus reduces food intake: neuroanatomical, electrophysiological, and behavioral evidence. Endocrinology 155(11):4356–4367 25116706 10.1210/en.2014-1248 PMC4256827

[86] Swick JC et al (2015) Parabrachial nucleus contributions to glucagon-like peptide-1 receptor agonist-induced hypophagia. Neuropsychopharmacology 40(8):2001–2014 25703200 10.1038/npp.2015.50 PMC4839524

[87] Nardone S et al (2024) A spatially-resolved transcriptional atlas of the murine dorsal pons at single-cell resolution. Nat Commun 15(1):1966 38438345 10.1038/s41467-024-45907-7 PMC10912765

[88] Siletti K et al (2023) Transcriptomic diversity of cell types across the adult human brain. Sci 382(6667):7046 10.1126/science.add7046 37824663

[89] Coulter AA, Rebello CJ, Greenway FL (2018) Centrally acting agents for obesity: past, present, and future. Drugs 78(11):1113–1132 30014268 10.1007/s40265-018-0946-y PMC6095132

[90] Sciolino NR et al (2022) Natural locus coeruleus dynamics during feeding. Sci Adv 8(33):9134 10.1126/sciadv.abn9134 PMC9390985 35984878

[91] Fortin SM et al (2023) The locus coeruleus contributes to the anorectic, nausea, and autonomic physiological effects of glucagon-like peptide-1. Sci Adv 9(38):0980 10.1126/sciadv.adh0980 PMC10511187 37729419

[92] Reiner DJ et al (2018) Glucagon-like peptide-1 receptor signaling in the lateral dorsal tegmental nucleus regulates energy balance. Neuropsychopharmacology 43(3):627–637 28920591 10.1038/npp.2017.225 PMC5770766

[93] Salamone JD et al (2016) Mesolimbic dopamine and the regulation of motivated behavior. Curr Top Behav Neurosci 27:231–257 26323245 10.1007/7854_2015_383

[94] Adamantidis AR et al (2011) Optogenetic interrogation of dopaminergic modulation of the multiple phases of reward-seeking behavior. J Neurosci 31(30):10829–10835 21795535 10.1523/JNEUROSCI.2246-11.2011 PMC3171183

[95] van Zessen R et al (2012) Activation of VTA GABA neurons disrupts reward consumption. Neuron 73(6):1184–1194 22445345 10.1016/j.neuron.2012.02.016 PMC3314244

[96] Fernandes AB et al (2020) Postingestive modulation of food seeking depends on vagus-mediated dopamine neuron activity. Neuron 106(5):778–788 32259476 10.1016/j.neuron.2020.03.009 PMC7710496

[97] Wang XF et al (2015) Endogenous glucagon-like peptide-1 suppresses high-fat food intake by reducing synaptic drive onto mesolimbic dopamine neurons. Cell Rep 12(5):726–733 26212334 10.1016/j.celrep.2015.06.062 PMC4860285

[98] Alhadeff AL, Rupprecht LE, Hayes MR (2012) GLP-1 neurons in the nucleus of the solitary tract project directly to the ventral tegmental area and nucleus accumbens to control for food intake. Endocrinology 153(2):647–658 22128031 10.1210/en.2011-1443 PMC3275387

[99] Mietlicki-Baase EG et al (2013) The food intake-suppressive effects of glucagon-like peptide-1 receptor signaling in the ventral tegmental area are mediated by AMPA/kainate receptors. Am J Physiol Endocrinol Metab 305(11):E1367–E1374 24105414 10.1152/ajpendo.00413.2013 PMC3882373

[100] Dickson SL et al (2012) The glucagon-like peptide 1 (GLP-1) analogue, exendin-4, decreases the rewarding value of food: a new role for mesolimbic GLP-1 receptors. J Neurosci 32(14):4812–4820 22492036 10.1523/JNEUROSCI.6326-11.2012 PMC6620919

[101] Falk S et al (2023) GLP-1 and nicotine combination therapy engages hypothalamic and mesolimbic pathways to reverse obesity. Cell Rep 42(5):112466 37148870 10.1016/j.celrep.2023.112466

[102] Dossat AM et al (2011) Glucagon-like peptide 1 receptors in nucleus accumbens affect food intake. J Neurosci 31(41):14453–14457 21994361 10.1523/JNEUROSCI.3262-11.2011 PMC3328130

[103] Mietlicki-Baase EG et al (2014) Glucagon-like peptide-1 receptor activation in the nucleus accumbens core suppresses feeding by increasing glutamatergic AMPA/kainate signaling. J Neurosci 34(20):6985–6992 24828651 10.1523/JNEUROSCI.0115-14.2014 PMC4019807

[104] Tellez LA et al (2013) A gut lipid messenger links excess dietary fat to dopamine deficiency. Science 341(6147):800–802 23950538 10.1126/science.1239275

[105] Hankir MK et al (2017) Gastric bypass surgery recruits a gut PPAR-alpha-striatal D1R pathway to reduce fat appetite in obese rats. Cell Metab 25(2):335–344 28065827 10.1016/j.cmet.2016.12.006

[106] Han W et al (2018) A neural circuit for gut-induced reward. Cell 175(3):887–888 30340046 10.1016/j.cell.2018.10.018

[107] Garfield AS, Heisler LK (2009) Pharmacological targeting of the serotonergic system for the treatment of obesity. J Physiol 587(1):49–60 19029184 10.1113/jphysiol.2008.164152 PMC2670022

[108] Nectow AR et al (2017) Identification of a brainstem circuit controlling feeding. Cell 170(3):429–442 28753423 10.1016/j.cell.2017.06.045

[109] Anderberg RH et al (2017) Glucagon-like peptide 1 and its analogs act in the dorsal raphe and modulate central serotonin to reduce appetite and body weight. Diabetes 66(4):1062–1073 28057699 10.2337/db16-0755 PMC6237271

[110] Asarian L (2009) Loss of cholecystokinin and glucagon-like peptide-1-induced satiation in mice lacking serotonin 2C receptors. Am J Physiol Regul Integr Comp Physiol 296(1):R51–R56 19005016 10.1152/ajpregu.90655.2008

[111] Nonogaki K et al (2011) The contribution of serotonin 5-HT2C and melanocortin-4 receptors to the satiety signaling of glucagon-like peptide 1 and liraglutide, a glucagon-like peptide 1 receptor agonist, in mice. Biochem Biophys Res Commun 411(2):445–448 21756875 10.1016/j.bbrc.2011.06.175

[112] Ratner C et al (2012) Cerebral markers of the serotonergic system in rat models of obesity and after Roux-en-Y gastric bypass. Obesity (Silver Spring) 20(10):2133–2141 22450706 10.1038/oby.2012.75 PMC3562999

[113] Tovote P, Fadok JP, Luthi A (2015) Neuronal circuits for fear and anxiety. Nat Rev Neurosci 16(6):317–331 25991441 10.1038/nrn3945

[114] Hao S et al (2019) The lateral hypothalamic and BNST GABAergic projections to the anterior ventrolateral periaqueductal gray regulate feeding. Cell Rep 28(3):616–624 31315042 10.1016/j.celrep.2019.06.051

[115] Wang X et al (2023) Neural adaption in midbrain GABAergic cells contributes to high-fat diet-induced obesity. Sci Adv 9(44):2884 10.1126/sciadv.adh2884 PMC10619925 37910621

[116] Anand BK, Brobeck JR (1951) Localization of a “feeding center” in the hypothalamus of the rat. Proc Soc Exp Biol Med 77(2):323–324 14854036 10.3181/00379727-77-18766

[117] Hetherington AW, Ranson SW (1940) Hypothalamic lesions and adiposity in the rat. Anat Rec 78(2):149–172

[118] Katsurada K et al (2014) Endogenous GLP-1 acts on paraventricular nucleus to suppress feeding: projection from nucleus tractus solitarius and activation of corticotropin-releasing hormone, nesfatin-1 and oxytocin neurons. Biochem Biophys Res Commun 451(2):276–281 25089000 10.1016/j.bbrc.2014.07.116

[119] Maejima Y et al (2021) The deletion of glucagon-like peptide-1 receptors expressing neurons in the dorsomedial hypothalamic nucleus disrupts the diurnal feeding pattern and induces hyperphagia and obesity. Nutr Metab (Lond) 18(1):58 34098999 10.1186/s12986-021-00582-z PMC8186199

[120] Lopez-Ferreras L et al (2018) Lateral hypothalamic GLP-1 receptors are critical for the control of food reinforcement, ingestive behavior and body weight. Mol Psychiatry 23(5):1157–1168 28894301 10.1038/mp.2017.187 PMC5984105

[121] Norsted E, Gomuc B, Meister B (2008) Protein components of the blood-brain barrier (BBB) in the mediobasal hypothalamus. J Chem Neuroanat 36(2):107–121 18602987 10.1016/j.jchemneu.2008.06.002

[122] Ciofi P et al (2009) Brain-endocrine interactions: a microvascular route in the mediobasal hypothalamus. Endocrinology 150(12):5509–5519 19837874 10.1210/en.2009-0584 PMC2819742

[123] Balland E et al (2014) Hypothalamic tanycytes are an ERK-gated conduit for leptin into the brain. Cell Metab 19(2):293–301 24506870 10.1016/j.cmet.2013.12.015 PMC3936883

[124] Duquenne M et al (2021) Leptin brain entry via a tanycytic LepR-EGFR shuttle controls lipid metabolism and pancreas function. Nat Metab 3(8):1071–1090 34341568 10.1038/s42255-021-00432-5 PMC7611554

[125] Imbernon M et al (2022) Tanycytes control hypothalamic liraglutide uptake and its anti-obesity actions. Cell Metab 34(7):1054–1063 35716660 10.1016/j.cmet.2022.06.002 PMC7613793

[126] Secher A et al (2014) The arcuate nucleus mediates GLP-1 receptor agonist liraglutide-dependent weight loss. J Clin Invest 124(10):4473–4488 25202980 10.1172/JCI75276 PMC4215190

[127] Beiroa D et al (2014) GLP-1 agonism stimulates brown adipose tissue thermogenesis and browning through hypothalamic AMPK. Diabetes 63(10):3346–3358 24917578 10.2337/db14-0302

[128] Burmeister MA et al (2017) The hypothalamic glucagon-like peptide 1 receptor is sufficient but not necessary for the regulation of energy balance and glucose homeostasis in mice. Diabetes 66(2):372–384 27908915 10.2337/db16-1102 PMC5248999

[129] Kim KS et al (2024) GLP-1 increases preingestive satiation via hypothalamic circuits in mice and humans. Sci 2537 10.1126/science.adj2537 PMC11961025 38935778

[130] Chen Z et al (2024) GLP-1R-positive neurons in the lateral septum mediate the anorectic and weight-lowering effects of liraglutide in mice. J Clin Invest 134(17) 10.1172/JCI178239 PMC11364389 39225090

[131] Ghosal S et al (2017) Disruption of glucagon-like peptide 1 signaling in Sim1 neurons reduces physiological and behavioral reactivity to acute and chronic stress. J Neurosci 37(1):184–193 28053040 10.1523/JNEUROSCI.1104-16.2016 PMC5214629

[132] Burmeister MA et al (2017) The glucagon-like peptide-1 receptor in the ventromedial hypothalamus reduces short-term food intake in male mice by regulating nutrient sensor activity. Am J Physiol Endocrinol Metab 313(6):E651–E662 28811293 10.1152/ajpendo.00113.2017 PMC6109646

[133] Hahn TM et al (1998) Coexpression of Agrp and NPY in fasting-activated hypothalamic neurons. Nat Neurosci 1(4):271–272 10195157 10.1038/1082

[134] Aponte Y, Atasoy D, Sternson SM (2011) AGRP neurons are sufficient to orchestrate feeding behavior rapidly and without training. Nat Neurosci 14(3):351–355 21209617 10.1038/nn.2739 PMC3049940

[135] Atasoy D et al (2012) Deconstruction of a neural circuit for hunger. Nature 488(7410):172–177 22801496 10.1038/nature11270 PMC3416931

[136] Betley JN et al (2015) Neurons for hunger and thirst transmit a negative-valence teaching signal. Nature 521(7551):180–185 25915020 10.1038/nature14416 PMC4567040

[137] Krashes MJ et al (2011) Rapid, reversible activation of AgRP neurons drives feeding behavior in mice. J Clin Invest 121(4):1424–1428 21364278 10.1172/JCI46229 PMC3069789

[138] Steculorum SM et al (2016) AgRP neurons control systemic insulin sensitivity via myostatin expression in brown adipose tissue. Cell 165(1):125–138 27015310 10.1016/j.cell.2016.02.044 PMC5157157

[139] Li H et al (2023) The melanocortin action is biased toward protection from weight loss in mice. Nat Commun 14(1):2200 37069175 10.1038/s41467-023-37912-z PMC10110624

[140] Zhu C et al (2020) Profound and redundant functions of arcuate neurons in obesity development. Nat Metab 2(8):763–774 32719538 10.1038/s42255-020-0229-2 PMC7687864

[141] Ewbank SN et al (2020) Chronic G(q) signaling in AgRP neurons does not cause obesity. Proc Natl Acad Sci U S A 117(34):20874–20880 32764144 10.1073/pnas.2004941117 PMC7456117

[142] Cai J et al (2023) AgRP neurons are not indispensable for body weight maintenance in adult mice. Cell Rep 42(7):112789 37422762 10.1016/j.celrep.2023.112789 PMC10909125

[143] De Solis AJ et al (2024) Reciprocal activity of AgRP and POMC neurons governs coordinated control of feeding and metabolism. Nat Metab 6(3):473–493 38378998 10.1038/s42255-024-00987-z PMC10963273

[144] Chen Y et al (2016) Hunger neurons drive feeding through a sustained, positive reinforcement signal. Elife 5 10.7554/eLife.18640 PMC5016090 27554486

[145] Chen Y et al (2015) Sensory detection of food rapidly modulates arcuate feeding circuits. Cell 160(5):829–841 25703096 10.1016/j.cell.2015.01.033 PMC4373539

[146] Nakazato M et al (2001) A role for ghrelin in the central regulation of feeding. Nature 409(6817):194–198 11196643 10.1038/35051587

[147] Tschop M, Smiley DL, Heiman ML (2000) Ghrelin induces adiposity in rodents. Nature 407(6806):908–913 11057670 10.1038/35038090

[148] Beutler LR et al (2017) Dynamics of gut-brain communication underlying hunger. Neuron 96(2):461–475 29024666 10.1016/j.neuron.2017.09.043 PMC5691364

[149] Su Z, Alhadeff AL, Betley JN (2017) Nutritive, post-ingestive signals are the primary regulators of AgRP neuron activity. Cell Rep 21(10):2724–2736 29212021 10.1016/j.celrep.2017.11.036 PMC5724395

[150] Goldstein N et al (2021) Hypothalamic detection of macronutrients via multiple gut-brain pathways. Cell Metab 33(3):676–687 33450178 10.1016/j.cmet.2020.12.018 PMC7933100

[151] Campbell JN et al (2017) A molecular census of arcuate hypothalamus and median eminence cell types. Nat Neurosci 20(3):484–496 28166221 10.1038/nn.4495 PMC5323293

[152] Steuernagel L et al (2022) HypoMap-a unified single-cell gene expression atlas of the murine hypothalamus. Nat Metab 4(10):1402–1419 36266547 10.1038/s42255-022-00657-y PMC9584816

[153] Lam BYH et al (2017) Heterogeneity of hypothalamic pro-opiomelanocortin-expressing neurons revealed by single-cell RNA sequencing. Mol Metab 6(5):383–392 28462073 10.1016/j.molmet.2017.02.007 PMC5404100

[154] Biglari N et al (2021) Functionally distinct POMC-expressing neuron subpopulations in hypothalamus revealed by intersectional targeting. Nat Neurosci 24(7):913–929 34002087 10.1038/s41593-021-00854-0 PMC8249241

[155] Dong Y et al (2021) Time and metabolic state-dependent effects of GLP-1R agonists on NPY/AgRP and POMC neuronal activity in vivo. Mol Metab 54:101352 34626854 10.1016/j.molmet.2021.101352 PMC8590079

[156] Tan HL et al (2024) Leptin-activated hypothalamic BNC2 neurons acutely suppress food intake. Nat 10.1038/s41586-024-08108-2 PMC11618066 39478220

[157] Gantz I et al (1993) Molecular cloning, expression, and gene localization of a fourth melanocortin receptor. J Biol Chem 268(20):15174–15179 8392067

[158] Mul JD et al (2012) Effect of vertical sleeve gastrectomy in melanocortin receptor 4-deficient rats. Am J Physiol Endocrinol Metab 303(1):E103–E110 22535749 10.1152/ajpendo.00159.2012 PMC3404562

[159] Hatoum IJ et al (2012) Melanocortin-4 receptor signaling is required for weight loss after gastric bypass surgery. J Clin Endocrinol Metab 97(6):E1023–E1031 22492873 10.1210/jc.2011-3432 PMC3387412

[160] Garfield AS et al (2015) A neural basis for melanocortin-4 receptor-regulated appetite. Nat Neurosci 18(6):863–871 25915476 10.1038/nn.4011 PMC4446192

[161] Fratzl A, Hofer SB (2022) The caudal prethalamus: inhibitory switchboard for behavioral control? Neuron 110(17):2728–2742 36076337 10.1016/j.neuron.2022.07.018

[162] Zhang X, van den Pol AN (2017) Rapid binge-like eating and body weight gain driven by zona incerta GABA neuron activation. Science 356(6340):853–859 28546212 10.1126/science.aam7100 PMC6602535

[163] Ong ZY et al (2017) Paraventricular thalamic control of food intake and reward: role of glucagon-like peptide-1 receptor signaling. Neuropsychopharmacology 42(12):2387–2397 28811669 10.1038/npp.2017.150 PMC5645740

[164] Ye Q, Nunez J, Zhang X (2023) Zona incerta dopamine neurons encode motivational vigor in food seeking. Sci Adv 9(46):5326 10.1126/sciadv.adi5326 PMC10656063 37976360

[165] Terrill SJ et al (2016) Role of lateral septum glucagon-like peptide 1 receptors in food intake. Am J Physiol Regul Integr Comp Physiol 311(1):R124–R132 27194565 10.1152/ajpregu.00460.2015 PMC4967229

[166] Terrill SJ, Maske CB, Williams DL (2018) Endogenous GLP-1 in lateral septum contributes to stress-induced hypophagia. Physiol Behav 192:17–22 29510158 10.1016/j.physbeh.2018.03.001 PMC6019151

[167] Terrill SJ et al (2019) Endogenous GLP-1 in lateral septum promotes satiety and suppresses motivation for food in mice. Physiol Behav 206:191–199 30980855 10.1016/j.physbeh.2019.04.008 PMC6956655

[168] Lu Y et al (2024) Dorsolateral septum GLP-1R neurons regulate feeding via lateral hypothalamic projections. Mol Metab 85:101960 38763494 10.1016/j.molmet.2024.101960 PMC11153235

[169] Kanoski SE et al (2011) Hippocampal leptin signaling reduces food intake and modulates food-related memory processing. Neuropsychopharmacology 36(9):1859–1870 21544068 10.1038/npp.2011.70 PMC3154104

[170] Kanoski SE et al (2013) Ghrelin signaling in the ventral hippocampus stimulates learned and motivational aspects of feeding via PI3K-Akt signaling. Biol Psychiatry 73(9):915–923 22884970 10.1016/j.biopsych.2012.07.002 PMC3498600

[171] Hsu TM et al (2015) Hippocampal GLP-1 receptors influence food intake, meal size, and effort-based responding for food through volume transmission. Neuropsychopharmacology 40(2):327–337 25035078 10.1038/npp.2014.175 PMC4443945

[172] Hsu TM et al (2018) A hippocampus to prefrontal cortex neural pathway inhibits food motivation through glucagon-like peptide-1 signaling. Mol Psychiatry 23(7):1555–1565 28461695 10.1038/mp.2017.91 PMC5668211

[173] Hankir MK et al (2018) Brain feeding circuits after Roux-en-Y gastric bypass. Trends Endocrinol Metab 29(4):218–237 29475578 10.1016/j.tem.2018.01.009

[174] Miras AD et al (2019) Adjunctive liraglutide treatment in patients with persistent or recurrent type 2 diabetes after metabolic surgery (GRAVITAS): a randomised, double-blind, placebo-controlled trial. Lancet Diabetes Endocrinol 7(7):549–559 31174993 10.1016/S2213-8587(19)30157-3

[175] Mok J et al (2023) Safety and efficacy of liraglutide, 3.0 mg, once daily vs placebo in patients with poor weight loss following metabolic surgery: the BARI-OPTIMISE randomized clinical trial. JAMA Surg 158(10):1003–1011 10.1001/jamasurg.2023.2930 PMC10372755 37494014

